# Comparison of Conventional Oxygen Therapy With High-Flow Nasal Oxygenation in the Management of Hypercapnic Respiratory Failure

**DOI:** 10.7759/cureus.26815

**Published:** 2022-07-13

**Authors:** Jitendra Pratap Singh, Deepak Malviya, Samiksha Parashar, Soumya Sankar Nath, Archana Gautam, Neha Shrivastava

**Affiliations:** 1 Anaesthesiology and Critical Care, Dr. Ram Manohar Lohia Institute of Medical Sciences, Lucknow, IND

**Keywords:** hypercapnic respiratory failure, high-flow nasal cannula (hfnc), partial pressure of arterial oxygen, serum bicarbonate, non-invasive mechanical ventilation, partial pressure of arterial carbon dioxide

## Abstract

Introduction: The effectiveness of high-flow nasal oxygenation (HFNO) in patients with hypercapnic respiratory failure (RF) remains controversial. The current study compared the effectiveness of HFNO in patients with hypercapnic RF with conventional oxygen therapy (COT).

Objectives: The primary objective was to compare changes in the partial pressure of carbon dioxide (PaCO2) between those receiving COT and HFNO. The secondary objectives were to compare changes in the partial pressure of oxygen (PaO2), oxygen saturation (SpO2), respiratory rate (RR), serum bicarbonate level, base excess, lactate level, and incidence of the need for non-invasive ventilation (NIV) and mechanical ventilation (MV).

Methods: We recruited 30 patients with mild to moderate hypercapnic RF in the HFNO group, and data of 30 patients from historical controls, who matched the inclusion criteria, were obtained from medical records for comparison (COT group). The study was terminated after two hours, and patients were managed per the existing protocol after that. Arterial blood gas (ABG) analysis was repeated at the baseline, first, second, and third hours.

Results: In the COT group, the mean RR at the baseline, first, second, and third hours was 24.5 ± 2.61, 24.9 ± 3.03, 26.03 ± 3.4, and 22.90 ± 1.86, whereas, in the HFNO group, it was 25.93 ± 3.91, 23.00 ± 3.54, 22.50 ± 3.38, and 21.90 ± 3.57, respectively. The mean PaCO2 in the COT vs. HFNO groups was 54.45 ± 5.83 vs. 62.22 ± 9.15, 57.74 ± 6.05 vs. 58.65 ± 10.43, 60.79 ± 7.48 vs. 60.41 ± 11.24, and 55.23 ± 6.63 vs. 56.95 ± 10.31. The mean SpO2 in the COT group at these points of time was 94.50 ± 1.46, 95.4 ± 1.28, 96.10 ± 1.84, and 97.53 ± 2.05, whereas, in the HFNO group, it was 95.40 ± 2.55, 98.63 ± 1.43, 99.00 ± 1.66, and 99.50 ± 1.31, respectively. The patients who needed NIV after the study period were 50% and 36.67% in the COT and HFNO groups, respectively.

Conclusions: There was no change in PaCO2 levels with HFNO, but there was a significant improvement in SpO2 and PaO2 levels and a decreased RR. Following the termination of the study protocol, more patients in the COT group needed NIV than those in the HFNO group.

## Introduction

High-flow nasal oxygenation (HFNO) is a non-invasive, easy-to-use respiratory device, which displays several advantages over conventional oxygenation systems [1]. It can deliver warm, humidified flow up to 60 L/minute through a soft nasal cannula. The active humidification and warming of the delivered air-oxygen mixture promotes physiologic mucociliary function (like trapping several bacteria and clearance of secretions), ensures fluidity of respiratory secretions, and enables the patients to tolerate high flows [2,3]. Further, it has been seen that the fraction of inspired oxygen (FiO2) delivered to the patient remains stable and reliable as there are minimal losses and negligible ambient air entrainment [2,3]. HFNO has been increasingly used in patients with acute hypoxemic (type I) respiratory failure (RF). However, indications and clinical effectiveness of HFNO in patients with hypercapnic (type II) RF remain controversial. In hypercapnic RF, HFNO has been shown to cause at least two predominant effects; first, it generates a wash-out effect on the upper airway, which promotes carbon dioxide (CO2) elimination [4]; second, it generates positive end-expiratory pressure (PEEP) of up to 6 cm of H2O in the airway [5].

Despite these supposed advantages, current data on the uses of HFNO in hypercapnic patients with RF are ambiguous. Until now, very few reports, retrospective studies [5], studies that involved stable outdoor patients of chronic obstructive airway disease (COAD) [6], and trials that used HFNO for a very short period [7,8] were reported.

The present study was designed to evaluate the effectiveness of HFNO in patients of COAD with predominantly type II RF requiring ICU admission. The current study aims to compare the effectiveness of HFNO in patients with hypercapnic RF with conventional oxygen therapy (COT). The primary objective of the current study was to compare changes in the partial pressure of carbon dioxide (PaCO2) between those receiving COT and HFNO. The secondary objectives were to compare changes in the partial pressure of oxygen (PaO2), peripheral oxygen saturation (SpO2), respiratory rate (RR), serum bicarbonate (HCO3), base excess (BE), lactate, and incidence of the need for non-invasive ventilation (NIV) and mechanical ventilation (MV) between the two groups.

## Materials and methods

This prospective, comparative, observational study commenced after approval from the Institutional Ethics Committee, Dr. Ram Manohar Lohia Institute of Medical Sciences, Lucknow (IEC number: 67/19; reference number: 5172/RMLIMS/19). The study was registered with Clinical Trials Registry India (CTRI/2021/09/036515; dated: September 15, 2021). This study was conducted in the intensive care unit of a tertiary care hospital. Written informed consent was obtained from the patients or their next of kin before enrolling in the study. Patients were free to withdraw from the study at any time without adversely influencing their treatment. Patients with known COAD admitted because of predominant hypercapnic RF and fulfilling the inclusion criteria were enrolled in the study.

Calculation of sample size

A previous study showed that HFNO causes a decrease in PaCO2 of 4.2 mmHg [4]. The sample size of 25 will have 80% power, with a type I error rate of 5%, to detect a difference in PaCO2 of 4.2 mmHg [6]. So, we recruited 30 patients with mild to moderate hypercapnic RF in the HFNO group. Data of 30 patients from historical controls, who matched inclusion criteria, were obtained from medical records for comparison (COT group). Patients in the COT group received oxygen through a venturi mask with a FiO2, which ensured SpO2 between 89% and 94%.

Diagnosis and grading of mild and moderate chronic obstructive pulmonary disease (COPD) were made based on the Global Initiative for Chronic Obstructive Lung Disease (GOLD) criteria (GOLD 1 and GOLD 2), Modified Medical Research Council Dyspnea Scale, and COPD Assessment Test [9]. Hypercapnia was defined by PaCO2 ≥ 45 mmHg. Mild to moderate type 2 RF is defined as the patient receiving 4 liter/minute oxygen via nasal prongs for more than four hours and/or RR of 25 breaths/minute and/or increased work of breathing, evidenced by clinical signs such as dyspnea, in-drawing of the chest wall, accessory-muscle use, and diaphoresis or those receiving 6 liter/minute oxygen via a face mask for more than two hours and/or RR of 25 breaths/minute and/or increased work of breathing, evidenced by clinical signs such as dyspnea, retraction of the chest wall, accessory-muscle use, and diaphoresis along with PaCO2 > 45 mmHg. Patients requiring imminent mechanical ventilation were excluded.

We recorded demographic data, arterial blood gas (ABG) values (pH, PaCO2, PaO2, HCO3, BE, and serum lactate), SpO2, RR, blood pressure, heart rate, Glasgow Coma Scale (GCS) score, and chest auscultation findings (presence of rhonchi and/or crepitations) at baseline (zero hours), and then at first and second hours, after instituting HFNO therapy. The study was terminated after two hours, and after that, the patients were managed as per the existing protocol. One more ABG was repeated in the third hour, i.e. one hour after study termination, to assess the impact of change in therapy. Vital parameters were recorded on the Dräger Infinity C700 monitor with Infinity Kappa System, manufactured by Dräger Medical Systems Inc., Houston, TX.

Participants received high-flow oxygen via HFNO mode on Skanray CV200 ventilator (Bharat Electronics Ltd., Bengaluru, India) with MR850 humidifier, RT241 heated delivery tube, and RT033 large/RT034 small, wide-bore nasal cannula manufactured by Fisher & Paykel Healthcare, Auckland, New Zealand. HFNO was started with 0.25 liter/kg ideal body weight and increased to 1 liter/kg ideal body weight. Blended humidified oxygen was heated to 37°C before commencing HFNO, and flow was titrated according to patient comfort and SpO2.

After seeking consent and a negative Allen test, the radial artery was cannulated after infiltration of local anesthesia (2% lignocaine). ABG analysis was done on Siemens RAPIDLab 1200 Systems (Siemens Healthcare Diagnostics Ltd., Surrey, UK).

Inclusion criteria

Consenting adult patients (between 40 and 90 years) with COAD, with mild to moderate hypercapnic RF, admitted to our ICU were recruited for the study in the HFNO group.

Exclusion criteria

Patients who had co-existing hypoxemia (PaO2/FiO2 < 200), or were unconscious, unable to maintain airway because of any neurological reasons, hemodynamically unstable, or with a nasal condition affecting the ability to use a high-flow nasal cannula, e.g. nasal blockage, perforated nasal septum, and recent epistaxis, were excluded from the study.

Statistical analysis

Data are expressed as mean and standard deviation (SD) or median, range, and percentage as appropriate. All categorical data were compared by using the chi-square test. Continuous variables in two groups were compared by t-test. One-way ANOVA analyzed more than two variables. P-value < 0.05 was considered significant. Statistical analysis was done using Statistical Package for the Social Sciences (IBM SPSS Statistics for Windows, version 21.0, IBM Corp., Armonk, NY).

## Results

Table 1 shows the comparison of age, gender, weight, height, and smoking status among patients in the COT and HFNO groups, and all these parameters were comparable between the two groups.

**Table 1:** Comparison of demographic data between the two groups COT: conventional oxygen therapy; HFNO: high-flow nasal oxygenation.

S. No.	Parameter	COT (n = 30)	HFNO (n = 30)	P-value
1.	Age (mean ± SD) years	65.37 ± 9.67	67.21 ± 9.67	0.683
2.	Gender (male and female)	19 and 11	17 and 13	0.792
3.	Height (mean ± SD) cm	164.87 ± 10.33	164.61 ± 60.97	0.916
4.	Weight (mean ± SD) kg	64 ± 9.15	60.97 ± 11.40	0.164
5.	Smoking (yes and no)	22 and 8	19 and 11	0.579

Table 2 shows the comparison of RR, SpO2, PaCO2, PaO2, HCO3, BE, and serum lactate at the baseline, first, second, and third hours between the COT and HFNO groups. The RR was comparable at the baseline and first hour but significantly lower in the HFNO group at the second hour. Compared to COT, patients in the HFNO group had significantly higher SpO2 in the first, second, and third hours. Although the mean PaCO2 was significantly higher at the baseline in the HFNO group, it was comparable in both groups at the first, second, and third hours. The mean PaO2 was comparable at the baseline but significantly higher in patients of the HFNO group in the first, second, and third hours. The difference in HCO3 was significant at the baseline, but it was comparable between the groups in the first, second, and third hours. The mean BE and serum lactate levels were comparable between the two groups at all periods.

**Table 2:** Comparison of respiratory rate, blood gas parameters, and serum lactate at various times between the two groups * Statistically significant. COT: conventional oxygen therapy; HFNO: high-flow nasal oxygenation; SPO2: peripheral oxygen saturation; PaCO2: partial pressure of carbon dioxide; PaO2: partial pressure of oxygen; HCO3: serum arterial bicarbonate; BE: base excess.

	Baseline (mean ± SD)			1st hour (mean ± SD)			2nd hour (mean ± SD)			3rd hour (mean ± SD)		
	COT	HFNO	P-value	COT	HFNO	P-value	COT	HFNO	P-value	COT	HFNO	P-value
Respiratory rate (per minute)	24.5 ± 2.61	25.93 ± 3.91	0.100	24.90 ± 3.03	23.00 ± 3.54	0.030	26.03 ± 3.40	22.50 ± 3.38	<0.001	22.90 ± 1.86	21.90 ± 3.57	0.179
SpO2 (%)	94.50 ± 1.46	95.40 ± 2.55	0.099	95.40 ± 1.28	98.63 ± 1.43	<0.001*	96.10 ± 1.84	99.00 ± 1.66	<0.001*	97.53 ± 2.05	99.5 ± 1.31	<0.001*
PaCO2 (mmHg)	54.45 ± 5.83	62.22 ± 9.15	<0.001*	57.74 ± 6.05	58.65 ± 10.43	0.682	60.79 ± 7.48	60.41 ± 11.24	0.876	55.23 ± 6.63	56.95 ± 10.31	0.444
PaO2 (mmHg)	86.00 ± 6.61	90.10 ± 17.44	0.233	89.45 ± 6.44	105.16 ± 20.15	<0.001*	91.87 ± 5.81	104.11 ± 20.13	0.002*	98.60 ± 8.02	108.27 ± 15.31	0.003*
HCO3(meq/L)	28.72 ± 4.67	32.62 ± 7.78	0.022*	29.42 ± 4.56	39.18 ± 9.19	0.181	29.45 ± 4.49	32.17 ± 7.43	0.092	29.77 ± 4.42	31.90 ± 7.38	0.181
BE (meq/L)	3.75 ± 5.04	6.34 ± 6.00	0.076	4.66 ± 4.72	6.72 ± 5.44	0.122	5.03 ± 4.68	7.39 ± 5.13	0.068	5.49 ± 5.39	7.11 ± 4.96	0.229
Serum lactate (mg/dl)	10.287 ± 5.102	6.737 ± 3.390	0.002*	10.520 ± 4.865	6.617 ± 3.341	0.001*	10.893 ± 4.028	6.953 ± 3.681	<0.001*	11.537 ± 3.678	7.037 ± 3.696	<0.001*

Table 3 shows the comparison of the need for NIV and MV among the patients after the end of the study period between the COT and HFNO groups. The percentage of patients who needed NIV support was higher in the COT group than in the HFNO group but was not statistically significant.

**Table 3:** Comparison of the need for non-invasive ventilation and mechanical ventilation after the end of the study period between the two groups COT: conventional oxygen therapy; HFNO: high-flow nasal oxygenation.

	COT group (n = 30)	HFNO group (n = 30)	P-value
	Number (%)	Number (%)	
Non-invasive ventilation (NIV)	15 (50)	11 (36.67)	0.144
Mechanical ventilation (MV)	1 (3.33)	1 (3.33)	1.00

Table 4 shows the comparison of the ABG parameters like pH, PaCO2, PaO2, and BE at the baseline, first, second, and third hours in the two groups. In the HFNO group, pH, PaCO2, and BE were comparable at all times. pH and PaCO2 also showed significant variations in the COT group from baseline. PaO2 increased significantly in both COT and HFNO groups.

**Table 4:** Comparison of different ABG parameters at baseline, first, second, and third hours among the two groups * Statistically significant. ABG: arterial blood gas; COT: conventional oxygen therapy; HFNO: high-flow nasal oxygenation; PCO2: partial pressure of carbon dioxide; PO2: partial pressure of oxygen; BE: base excess.

	Baseline (mean ± SD)	1st hour (mean ± SD)	2nd hour (mean ± SD)	3rdhour (mean ± SD)	P-value
COT group					
pH	7.37 ± 0.09	7.32 ± 0.07	7.31 ± 0.08	7.37 ± 0.07	0.003*
PCO2	54.45 ± 5.83	57.74 ± 6.05	60.79 ± 7.48	55.23 ± 6.63	0.001*
PO2	86.00 ± 6.61	89.45 ± 6.44	91.87 ± 5.81	98.60 ± 8.02	<0.001*
BE	3.75 ± 5.04	4.66 ± 4.72	5.03 ± 4.68	5.49 ± 5.39	0.579
HFNO group					
pH	7.33 ± 0.09	7.35 ± 0.09	7.37 ± 0.12	7.38 ± 0.07	0.130
PCO2	62.22 ± 9.15	58.65 ± 10.43	60.41 ± 11.24	56.95 ± 10.31	0.231
PO2	90.10 ± 17.44	105.16 ± 20.15	104.11 ± 20.13	108.27 ± 15.31	0.001*
BE	6.34 ± 6.00	6.72 ± 5.44	7.39 ± 5.13	7.11 ± 4.96	0.883

Table 5 shows the Tukey post hoc comparisons of ABG parameters like pH, PaCO2, PaO2, and BE between different time intervals among the COT and HFNO groups. It revealed that in the COT group, pH and PaCO2 both showed significant differences in the second hour when compared to the baseline and in the third hour when compared to the second hour.

**Table 5:** Tukey post hoc multiple comparisons of ABG parameters at first, second, and third hours in the two groups * Statistically significant. ABG: arterial blood gas; COT: conventional oxygen therapy; HFNO: high-flow nasal oxygenation; PCO2: partial pressure of carbon dioxide; PO2: partial pressure of oxygen; BE: base excess.

ABG parameters	Baseline vs. 1st hour		Baseline vs. 2nd hour		Baseline vs. 3rd hour		1st vs. 2nd hour		1st vs. 3rd hour		2ndvs. 3rd hour	
	Mean difference	P-value	Mean difference	P-value	Mean difference	P-value	Mean difference	P-value	Mean difference	P-value	Mean difference	P-value
COT group												
pH	0.043	0.152	0.057	0.030*	-0.007	0.984	0.014	0.908	-0.051	0.068	-0.064	0.011*
PCO2	-3.287	0.214	-6.340	0.002*	-0.773	0.968	-3.053	0.274	2.513	0.447	5.567	0.007*
PO2	-3.444	0.205	-5.871	0.006	-12.597	<0.001*	-2.427	0.509	-9.153	<0.001*	-6.727	0.001*
BE	-0.903	0.895	-1.279	0.751	-1.734	0.532	-0.376	0.991	-0.830	0.916	-0.454	0.985
HFNO group												
pH	-0.02	0.895	-0.04	0.431	-0.06	0.113	-0.02	0.849	-0.04	0.407	-0.02	0.879
PCO2	3.57	0.538	1.81	0.904	5.27	0.201	-1.76	0.911	1.70	0.920	3.46	0.566
PO2	-15.05	0.010*	-14.01	0.020*	-18.17	0.001*	1.04	0.996	-3.12	0.913	-4.16	0.817
BE	-0.38	0.993	-1.06	0.873	-0.78	0.945	-0.67	0.963	-0.39	0.992	0.28	0.997

## Discussion

HFNO is a novel means of oxygen therapy with a commendable tolerance profile. However, most studies on HFNO for acute RF have excluded patients with hypercapnia [10]. The use of HFNO in stable COAD patients has been shown to reduce PaCO2, increase tidal volume (TV), and decrease minute ventilation (MV) and RR [11]. Fricke et al. (2016) reported that HFNO could improve alveolar ventilation and reduce PaCO2 in patients with COAD by clearing anatomical dead space [12]. The humidification of inhaled gases counteracts the drying effect of high flow and has resulted in greater patient comfort, higher tolerance, and better mucociliary clearance compared with conventional oxygenation methods [13].

HFNO improves gas exchange and oxygenation in a flow-dependent manner. It forms positive airway pressure (1.5-7 cm H2O) through the resistance of the nasopharynx and airway to high-flow gas. It provides an increased FiO2 because higher flow rates can match or exceed the patient's peak inspiratory flow, preventing entrainment of atmospheric air. Nasal and oropharyngeal dead space is washed out with oxygen-rich gas. The flow of 35 liter/minute with mouth closure has been shown to create positive expiratory nasopharynx pressures of up to 5.3 cm H2O [14].

In the present study, we found that PaCO2 increased from baseline until the first and second hours in the COT group. After termination of the study, it decreased at the end of the third hour when patients were switched to other modes of therapy, and changes in mean PaCO2 were significant (Table 2). In the HFNO group, PaCO2 decreased in the first hour compared to baseline but increased between the first and second hours. After the termination of the study at the third hour, it decreased, but changes from baseline were not significant (Table 2). On comparing PaCO2 between the COT and HFNO groups at baseline, before the application of HFNO, the PaCO2 was found to be significantly higher in the HFNO group. However, it was comparable in both the groups during the first, second, and third hours (Table 2). Thus, despite initially higher PaCO2, HFNO does not lead to a further increase in PaCO2 in patients prone to hypercapnia. Similar results were obtained by Pisani et al. (2017), who investigated 14 patients with hypercapnic COAD, who underwent 30 minutes of HFNO trial with a flow rate of 30 liter/minute, and found that, compared to baseline, RR was significantly reduced in HFNO with mouth closed, but fall in PaCO2 was not significant [15].

We observed that the PaCO2 in the HFNO group decreased from baseline in the first hour but again increased in the second hour, which further decreased in the third hour after the termination of the study. However, changes in PaCO2 were insignificant compared to the baseline and COT groups (Tables 2, 5). Lee et al. (2019) studied patients treated with a high-flow nasal cannula (HFNC) for acute hypercapnic RF and reported reduced PaCO2 leading to normalization of pH over time in the hypercapnic group. PaCO2 levels changed over time, but the pattern of change differed between patient groups. PaCO2 levels decreased in the hypercapnia group but tended to increase in the non-hypercapnia group [8]. Şancı et al. (2020), in their retrospective study, analyzed 120 patients with acute RF. They reported a substantial improvement in SpO2 and PaO2 and a decrease in serum lactate following HFNO. However, there was no significant decrease in PaCO2 [16]. Subgroup analysis revealed that only those who had hypercapnia demonstrated a fall in the PaCO2 levels with the use of HFNO. In contrast, those with COPD without hypercapnia did not demonstrate a significant change in PaCO2 levels. [16] The results of the present study concur with the findings of the study by Şancı et al. (2020) [16]. In a meta-analysis performed by Duan et al. (2021), it was found that HFNO did not decrease PaCO2 in the HFNO group compared to control groups but decreased PaCO2 compared to long-term oxygen therapy. HFNC resulted in better PaCO2 than control interventions in hypoxemic patients but not in other patients. They concluded that HFNC did not significantly decrease PaCO2 in COPD patients, which is in concordance with the present study [17]. Jeong et al. (2015), in a retrospective chart review of 81 patients with RF who were treated with HFNO, found that PaCO2 decreased significantly in the hypercapnic group and observed a reduction in PaCO2 in both hypercapnic and non-hypercapnic groups. However, the reduction was significant only in the hypercapnic group [1]. In another retrospective study by Kim et al. (2018), patients with acute RF with hypercapnia who required HFNC oxygen therapy for hypoxemia were included and it was observed that, with HFNC therapy, PaCO2 decreased and resulted in significant improvement in hypercapnia. They concluded that HFNC oxygen therapy with sufficient FiO2 to maintain a normal PaO2 significantly reduced PaCO2 in acute hypercapnic RF [4]. In many studies, HFNO was shown to decrease the PaCO2 in hypercapnic patients. However, hypercapnia in COPD patients in chronic settings might not improve as much as acute hypercapnia, with a therapy session lasting only three hours. The rationale behind these findings needs further investigation for a better understanding and utilization of HFNO.

On comparing mean PaO2 at the baseline, first, second, and third hours between COT and HFNO groups, mean PaO2 was comparable at baseline but significantly increased at the first, second, and third hours in the HFNO group (Tables 2, 5). Vargas et al. (2015), in their prospective comparative study involving 12 acute hypoxemic RF patients, found that compared with COT, HFNC improved inspiratory effort and oxygenation. HFNC caused a significant reduction in median RR compared with a non-rebreathing mask [18].

In the present study, the mean RR was comparable at baseline and first hour but was significantly lower in the HFNO group at the second hour. The SpO2 was found to be comparable at baseline but was significantly higher in the HFNO group compared to the COT group at the first and third hours (p < 0.001) (Table 2). HFNO initiation after supplemental oxygen therapy improves oxygen saturation and PaO2 in acute RF. Jones et al. (2016) found that HFNO improves PaO2 better than supplementing oxygen with a face mask [19]. Similar observations were reported by Şancı et al. (2020) and Gedikloglu et al. (2019) [16,20].

In the present study, we observed that the difference in serum HCO3 was significant between the groups at baseline but comparable between the groups in the first, second, and third hours. BE values were comparable between the groups (Tables 2, 4, 5). Comparing the serum lactate values between the groups, the mean difference in lactate level was significantly lower in the HFNO group. Similar changes in serum HCO3 and lactate values were reported by Şancı et al. [16].

The need for NIV and MV at the end of study periods was compared between the COT and HFNO groups. Although more patients in the COT group needed NIV than the HFNO group, the difference was statistically insignificant. The need for MV was comparable between the groups (Table 3). Jones et al. (2016) also did not find any reduction in the need for MV in patients of the HFNO group compared to COT [19]. Ni et al. (2017) performed a review and meta-analysis and reported a lesser need for intubation and MV in patients in the HFNO group than in the COT group [21]. A review of the literature by Zhao et al. (2017) also revealed a lesser need for intubation and MV in HFNO group patients compared to the COT group, which contradicts the findings of the present study [22]. More randomized control trials are required to approve or disapprove these findings.

Limitations of the study

We understand that the present study suffers from several limitations like small sample size, use of historical controls for comparison, and a study period that lasted only two hours.

## Conclusions

On analyzing and comparing our data with the findings of other authors, we conclude that although there was no change in PaCO2 levels with HFNO, there was a significant improvement in SpO2 and PaO2 levels and a decrease in the RR. Following the termination of the study protocol, more patients in the COT group needed NIV than those in the HFNO group.
